# Cytotoxic CD8^+^ T cells in cancer and cancer immunotherapy

**DOI:** 10.1038/s41416-020-01048-4

**Published:** 2020-09-15

**Authors:** Hans Raskov, Adile Orhan, Jan Pravsgaard Christensen, Ismail Gögenur

**Affiliations:** 1 Center for Surgical Science, Zealand University Hospital, Køge, Denmark; 2 Department of Biomedical Sciences, University of Copenhagen, Copenhagen, Denmark; 3 Department of Immunology and Microbiology, University of Copenhagen, Copenhagen, Denmark; 4 Department of Clinical Medicine, University of Copenhagen, Copenhagen, Denmark

**Keywords:** Checkpoint signalling, Cancer immunotherapy, Checkpoint signalling, Cancer immunotherapy, Cancer microenvironment

## Abstract

The functions of, and interactions between, the innate and adaptive immune systems are vital for anticancer immunity. Cytotoxic T cells expressing cell-surface CD8 are the most powerful effectors in the anticancer immune response and form the backbone of current successful cancer immunotherapies. Immune-checkpoint inhibitors are designed to target immune-inhibitory receptors that function to regulate the immune response, whereas adoptive cell-transfer therapies use CD8^+^ T cells with genetically modified receptors—chimaeric antigen receptors—to specify and enhance CD8^+^ T-cell functionality. New generations of cytotoxic T cells with genetically modified or synthetic receptors are being developed and evaluated in clinical trials. Furthermore, combinatory regimens might optimise treatment effects and reduce adverse events. This review summarises advances in research on the most prominent immune effectors in cancer and cancer immunotherapy, cytotoxic T cells, and discusses possible implications for future cancer treatment.

## Background

The natural immune response relies on the interaction of adaptive and innate immunity systems and the synergy between them. The essential aspects of this response in terms of anticancer immunity are the surveillance, detection and destruction of neoplastic cells. However, despite meticulous immune surveillance, cancer might eventually gain a foothold, and most immune cells will polarise into pro-tumorigenic or anti-tumorigenic cells, depending on a multitude of activating and inhibitory signals derived from cancer cells and their microenvironment.^1^ The outcome of this balance is a strong predictor of clinical outcome, with the infiltration of immune cells into, or their exclusion from, a tumour also being critical to the efficacy of immunotherapy.^2^

Cytotoxic CD8^+^ T cells of the adaptive immune system are the most powerful effectors in the anticancer immune response and constitute the backbone of cancer immunotherapy. Immune-checkpoint inhibitors aim to block suppressive immune receptors and revitalise dysfunctional T cells, including CD8^+^ T cells, and adoptive cell transfer uses CD8^+^ T cells with genetically modified receptors (chimaeric antigen receptors [CARs]). Both approaches have had enormous impacts on the outcomes of various cancers;^3^ indeed, the field of immuno-oncology has revolutionised the treatment of cancer in terms of efficacy and personalised treatment options, as monotherapy or combinatorial regimens, and will continue to do so in the years to come. Nevertheless, any positive responses might not last, and the side effects could force treatment discontinuation, indicating the need to continuously improve treatment strategies. New pathways and immune checkpoints are being investigated,^4^ and new generations of CAR T-cell products are currently being evaluated in clinical trials. This review summarises advances in research into cytotoxic CD8^+^ T cells, the most prominent effector cell type in cancer and cancer immunotherapy.

## CD8^+^ T cells

**Fig. 1:**
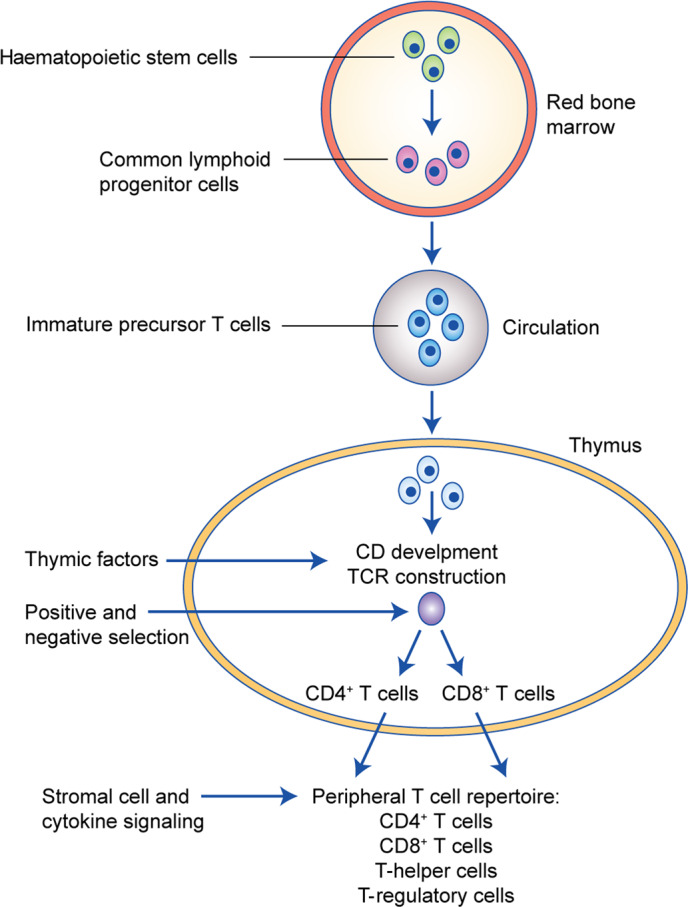
T-cell differentiation—an overview. Common lymphoid progenitor cells giving rise to immature precursor T cells originate in the red bone marrow. Due to the production in the thymus of chemotactic agents/thymic factors (e.g., thymotaxin, thymosin and thymopoietin), immature precursor T cells (being TCR- and CD-negative [double negative]) enter the circulation and are directed to the thymus. Within the thymus, the same agents induce the production of TCR and CD proteins. Thymic cells present the now CD- and TCR-positive T cells for MHC-1 and MHC-2 molecules to identify T-cell reactivity and direct their maturation pathways. During the positive selection process, T cells being able to bind MHC class I or II molecules with at least a weak affinity are identified. By negative selection, T cells with a high affinity for self-peptides undergo apoptosis to minimise the risk of immune responses towards self-proteins in the periphery. T cells with TCR affinity for MHC-1 become CD8^+^ T cells and T cells with TCR affinity for MHC-2 become CD4^+^ T cells. Depending on cytokine and stromal cell signalling, they may also differentiate into T-helper and T-regulatory cells. MHC: major histocompatibility complex.

T lymphocytes effectively navigate and scan almost all parts of the body for unwanted or foreign material; accordingly, naive and effector T cells are highly skilled migrators central to immune surveillance and the development of adaptive immunity against infection and cancer. Transcription factors, cytokines, chemokines, integrins and metabolic signals strictly regulate the differentiation and phenotypes of T cells, and T-cell lineages are considered to be fixed and mutually exclusive^5^ (Fig. 1). Cytotoxic CD8^+^ T cells are major killers of pathogens and neoplastic cells, with CD4^+^ T cells playing important roles in the maintenance of the CD8^+^ response and prevention of exhaustion.

### T-cell activation: initial interactions

CD8^+^ T cells interact with major histocompatibility complex class-1 (MHC-1) molecules^6^ on the surface of antigen-presenting cells (APCs) and target cells, which display antigenic peptide fragments produced by proteasomal degradation of cytoplasmic proteins bound to the corresponding binding grooves. MHC–antigen–peptide complexes are identified by CD8^+^ cells, which, upon engaging an APC or a target cell, attach to it and scan the surface by crawling over it. The direct contact and movements of the cells convert mechanical energy into biomechanical signals that play important roles in the activation of the CD8^+^ T-cell receptor (TCR) complex.^7^ By homing towards chemokine and integrin gradients on APCs or target cells, activated CD8^+^ T cells form immunological synapses between their supramolecular activation complex and adhesion molecules (such as intercellular adhesion molecule) on the target-cell surface.^8^ To confirm the nature of the target, the TCR and CD8, acting as a co-receptor, engage with the presented peptide and the MHC-α subunit, respectively. Following transduction of the TCR-activating signal, a co-stimulatory signal from the CD28 co-receptor must be received before the killing machinery is activated.

### T-cell activation: the TCR complex

**Fig. 2:**
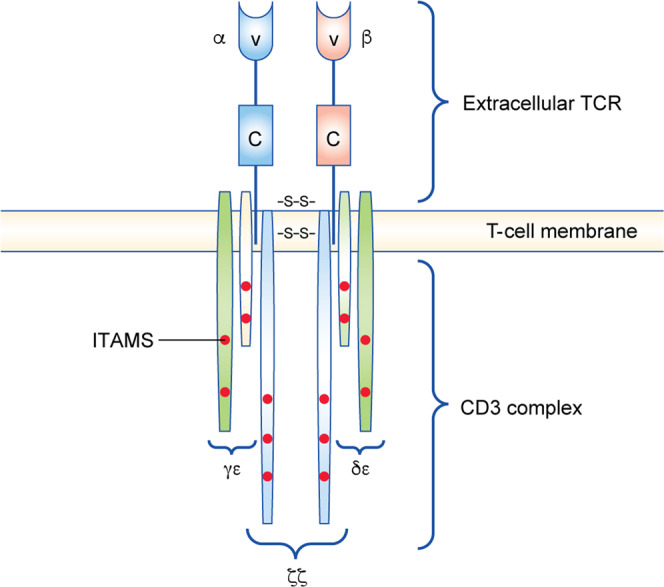
T-cell activation: the T-cell receptor (TCR) complex. Extracellularly, the TCR consists of the α and β chains, both of which have a constant region (C) and variable region (V), with the latter determining antigen specificity. The TCRαβ antigen-binding subunit is non-covalently bound to three CD3 co-receptor signalling subunits (ζζ, CD3δε and CD3γε), all of which contain immunoreceptor tyrosine-based activation motifs (ITAMs) in their cytoplasmic domains. SS disulfide bridge.

**Fig. 3:**
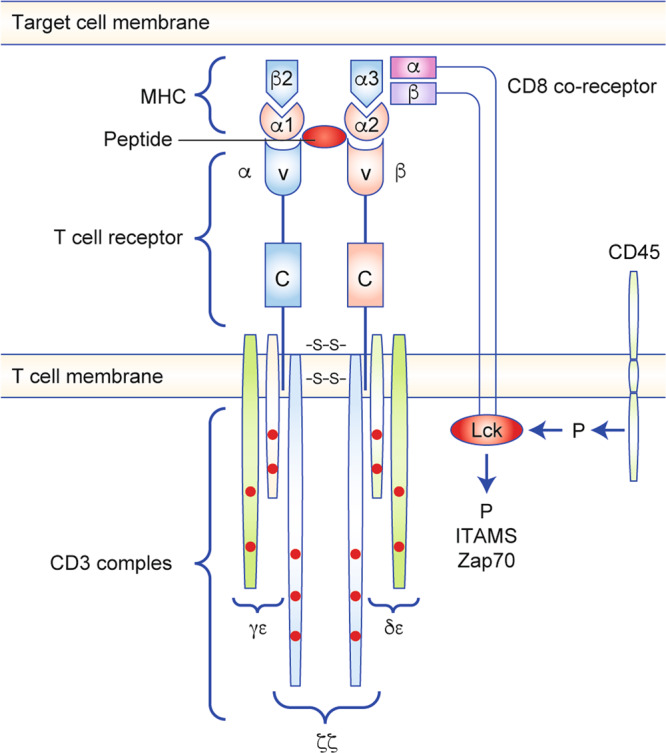
T-cell activation. The V domains of the α and β chains on T cells interact with an antigenic peptide presented by MHC-1 on the target cell, while the co-receptor CD8 associates with TCR–MHC-1 to tightly secure the TCR–CD3 complex to the major histocompatibility complex (MHC)–peptide complex. As CD8 binds to the MHC-1, Lck phosphorylates the intracellular portions of the CD3 ITAMs and positions ZAP-70 to phosphorylate the transmembrane proteins that allow the CD8^+^ T cell to secrete its cytokines. Lck: lymphocyte-specific protein tyrosine kinase, P: phosphorylation, CD45: receptor-linked protein tyrosine phosphatase, Zap70: ζ chain of T-cell-receptor-associated protein kinase 70.

The trigger mechanism for the formation of CD8^+^ TCR complex has not been mapped in detail. The TCR complex is an intricate structure, the aggregation of which involves a highly organised set of events. The TCR complex comprises the antigen-binding subunit (TCRαβ) non-covalently bound to three CD3 co-receptor signalling subunits (ζζ, CD3δε and CD3γε) (Fig. 2). The CD3 γ, δ, ε and ζ polypeptides all contain immunoreceptor tyrosine-based activation motifs (ITAMs) in their cytoplasmic domains, which are required for TCR surface expression, intracellular assembly and signal transduction.^9^ Both the TCRα and β chains contain a variable immunoglobulin-like domain (V domain) that determines antigen specificity, a constant domain (C domain), a membrane-connecting peptide, a transmembrane region and a short cytoplasmic tail that does not contain intracellular signalling motifs. Intracellular signalling is conducted by the CD3 complex, but requires CD8 for initiation and amplification (Fig. 3). CD45 is one of the most abundant cell-surface glycoproteins on T cells. It acts as a positive regulator of TCR signalling by dephosphorylating (and thereby activating) the kinase Lck through its intracellular tyrosine phosphatase domain; consequently, Lck can phosphorylate CD3 and the ζζ chains,^10^ thereby inducing downstream signalling.

The engagement of TCRαβ with an antigen peptide presented by the MHC class-1 protein on the target-cell surface leads to phosphorylation of the ITAMs in the intracellular domains of the signalling subunits.^11^ CD8, present as a co-receptor on the T-cell surface as an αα homodimer or an αβ heterodimer, associates with TCR–MHC-1 to tightly secure the TCR–CD3 complex to the MHC–peptide complex, and might increase sensitivity to peptide–MHC complexes by 100-fold.^12^ CD8 binds to the MHC on the α3 domain, which is separated from the TCR peptide α1- and α2- binding domains^13^ (Fig. 3), and is, as such, not an integrated part of the TCR complex; this is reflected by the fact that high-affinity TCRs are capable of CD8-independent interactions.^14^

### T-cell activation: the CD28 receptor

As described above, to become fully activated, the initial activating signal from the TCR signal must be followed by an independent, co-stimulatory signal. Without this co-stimulatory signal, CD8^+^ T cells become anergic and are directed to undergo apoptosis. This second signal is mediated by the interaction of CD28 receptors on CD8^+^ T cells with CD80/B7.1 or CD86/B7.2, both of which are highly expressed on APCs, macrophages and activated B cells, and plays a crucial role in determining CD8^+^ T-cell sensitivity^15^ by lowering the stimulation threshold of naive CD8^+^ T cells and enhancing cell proliferation and cytokine production (especially the production of interleukin [IL]-2). The second signal also protects CD8^+^ T cells from responding to self-antigens and thus decreases the risk of tissue damage and autoimmunity. Upon stimulation of the CD28 receptor, the intracellular tyrosine residues are phosphorylated, leading to the recruitment of phosphatidylinositol 3-kinase (PI3K). The CD28-mediated activation of PI3K promotes the activation of protein kinase B (PKB/Akt) and nuclear factor-κB (NF-κB), culminating in the upregulation of Bcl-xL expression and increased T-cell survival.^16^ As a consequence, the killing machinery can now be activated.

### Target-cell death

CD8^+^ T-cell–target-cell interactions are characterised by sustained motility of the CD8^+^ T cell on the target cell. As mentioned above, these mechanical forces can enhance pore formation in the target cell membrane and subsequent target-cell killing via secreted death-inducing granules containing granzymes, perforin, cathepsin C and granulysin fusing with the target-cell membrane.^17^ Alternatively, a complex of granulysin, perforin and granzymes is ingested by target cells through endocytosis of cytotoxic T-cell membranes. Granulysin and perforin subsequently create pores in the endosomal membrane and release several granzymes into the cytoplasm.^18^

In addition, Fas ligand (FASL) is expressed on CD8^+^ T cells and its ligation by Fas receptors on target cells activates death domains (Fas-associated protein with death domains [FADD]), which, in turn, activate caspases and endonucleases, leading to the fragmentation of target-cell DNA.^19^

### Overcoming target-cell death

Target-cell killing can take only a few minutes, and individual T cells are capable of carrying out serial or simultaneous killing of multiple target cells.^20^ However, cancer cells can develop defence mechanisms—for example, by downregulating the expression of MHC molecules and secreting perforin-degrading enzymes, as seen in melanoma cells.^21^ By contrast, in the case of an overactivated CD8^+^ T-cell response, tissue damage and autoimmunity might ensue,^22^ so, in order to maintain host self-tolerance and to avoid uncontrollable CD8^+^ T-cell activation, CD8^+^ T cells (and various other immune cells) transiently express immune-inhibitory receptors (known as immune-checkpoint molecules) to enable them to regulate the immune response appropriately in the context of the vast amount of incoming signals.^23^ However, a malignant tumour can exploit these signalling pathways to induce an immunosuppressive state that promotes its survival^24^ (see below).

Persistent exposure of CD8^+^ T cells to tumour neoantigens can induce the sustained expression of immune-checkpoint molecules, which characterises—and might drive—the dysfunctional state called T-cell exhaustion, in which simple removal of the antigen does not induce recovery.^25^ Exposure of CD8^+^ cells to antigens for only a few weeks led to an increased and sustained expression of cytotoxic T-lymphocyte-associated protein (CTLA-4) and subsequently to the development of inactive (exhausted) and apoptotic CD8^+^ T cells.^25^

Furthermore, exhausted CD8^+^ T cells retain their mitotic activity and contribute to creating or sustaining a suppressive environment.^26^

## Immune checkpoints

**Fig. 4:**
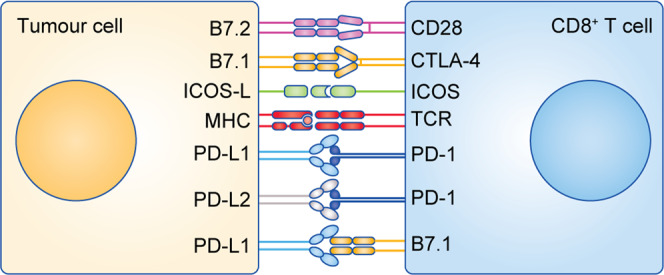
CD8^+^ T-cell immune-checkpoint receptors and their ligands. Receptors stimulating CD8^+^ T-cell functions include CD28, ICOS and B7.1. Receptors mediating inhibitory signals include CTLA-4, PD-1 and B7.1. CD28 and CTLA-4 compete for the ligands B7.1 and B7.2 during the early stages of the CD8^+^ T-cell response. pMHC: peptide-loaded major histocompatibility complex, ICOS: inducible T-cell co-stimulator.

Immune-checkpoint molecules are inhibitory receptors on the surface of immune cells (Fig. 4) that ensure appropriate regulation of the immune response. Prominent checkpoint molecules include programmed cell death receptor 1 (PD-1 or CD279) and CTLA-4 expressed by T cells, natural killer (NK) cells and activated macrophages. Other checkpoint molecules include lymphocyte-activation gene 3 (LAG-3), T-cell immunoglobulin and mucin domain-3 (TIM-3), T-cell immunoreceptor with Ig and ITIM domains (TIGIT) and inducible T-cell co-stimulatory receptor (ICOS) (reviewed by Joller et al.^27^ and Amatore et al.^28^) The expression of immune-checkpoint molecules can be used to monitor CD8^+^ T-cell exhaustion, as well as being used in combination with other markers, such as microsatellite instability, mismatch repair deficiency and the mutational burden, as positive predictive biomarkers for the efficacy of immunotherapy.^29^

As well as binding to CD28, as outlined above, B7 proteins on the surface of tumour cells constitute ligands for immune-checkpoint molecules: the PD-1 receptor ligands programmed death ligand 1 (PD-L1, also known as B7–H1/CD274) and programmed death ligand 2 (PD-L2, also known as B7-DC/CD273), and CTLA-4 ligands CD80 (B7.1) and CD86 (B7.2) activate immune checkpoints in T cells. The affinity of B7 proteins for CTLA-4 is much higher than for CD28, and for the tumour cell expressing these proteins, the net result is lymphocyte anergy and apoptosis.^30^

### The PD-1 checkpoint

PD-1 is a receptor that mediates immune-inhibitory signals through ligand binding by PD-L1 and PD-L2, which are frequently overexpressed on the surface of tumour cells. The PD-L1–PD-L2–PD-1 interaction antagonises CD80–CD28 co-stimulation and strongly counteracts TCR signal transduction, even at very low PD-1 expression levels through several poorly understood molecular mechanisms. The result is the abrogation of cytokine production by, and cycle arrest and decreased transcription of the pro-survival factor Bcl-X_L_ in, the cytotoxic CD8^+^ T cells.^31^ Furthermore, PD-1 engagement causes a rapid shift in CD8^+^ T-cell metabolism from glycolysis (which provides the fast energy increase that activation requires) towards fatty acid β-oxidation, leading to the accumulation of reactive oxygen species, mitochondrial damage and cell death.^32^ Potentiating TCR signalling by antibody blockade of PD-1 thus restores CD8^+^ T-cell functions.^31^

### The CTLA-4 checkpoint

The CTLA-4 receptor is considered the gatekeeper among immune checkpoints. CTLA-4 receptors are bound by the same ligands as CD28, but with a 20-times higher affinity, and thus outcompete CD28 for ligands. Following CD8^+^ T-cell activation, the expression of the CTLA-4 receptor is upregulated to transmit downstream-inhibitory signals to balance inputs and ensure that CD8^+^ T-cell activation does not become uncontrolled. CTLA-4 activation interferes with CD8^+^ T-cell movements and the ability to form stable conjugates with APCs, thus reducing the contact time between cells.^33^

While PD-1 signals are effective during the effector phase and predominantly occur within the peripheral tissues,^34^ CTLA-4 signals are effective during the priming phase of naive T-cell activation and primarily occur in lymphatic tissue. CTLA-4-knockout mice are unable to stop immune responses and develop fatal massive lymphocyte proliferation.^35^

## Immune-checkpoint inhibitor therapy

The introduction of monoclonal antibodies that target immune-inhibitory receptors, known as checkpoint inhibitors, has been an immense breakthrough in immuno-oncology, and has greatly improved the clinical outcomes of several cancers.^36^ This therapeutic strategy can enhance the efficacy of antitumour-immune responses, as well as revitalising exhausted CD8^+^ T cells. No other immunotherapy achieves the same degree of tumour cell killing,^37^ and anti-PD-1 agents in particular have revolutionised the treatment of metastatic melanoma, with durable responses occurring in more than 50% of patients surviving for the past 4 years.^38^ Between March 2011 and August 2018, six checkpoint inhibitors targeting the PD-1 pathway were approved for the treatment of 14 indications in the United States (three PD-1 inhibitors—pembrolizumab, nivolumab and cemiplimab, and three PD-L1 inhibitors—atezolizumab, avelumab and durvalumab).

For patients with complete responses, anti-PD-1 treatment has been shown to be able to induce a complete response in as few as 80 days.^39^ However, predicting tumour responses to PD-1 blockade is challenging. Evaluation of PD-L1 expression by immunohistochemistry using different assays and thresholds for PD-L1 expression^40^ is an approved method to guide treatment decisions in various cancers. It is clear that the presence of infiltrating CD8^+^ T cells in combination with increased PD-L1 expression/amplification is positively associated with the therapeutic efficacy of PD-1 blockade, although patients with PD-L1-negative tumours might also respond to treatment.^41^

However, not all patients with cancer display positive predictive biomarkers and achieve durable benefits. Indeed, a cross-sectional analysis found that the percentage of US cancer patients eligible for treatment with checkpoint inhibitor drugs (i.e., those patients who might benefit from and respond to these drugs, depending on their tumour type and levels of checkpoint molecules) was 43% in 2018, with only 13% of patients responding to these drugs.^42^ Furthermore, checkpoint inhibition is associated with a spectrum of gastrointestinal, dermatological, endocrine and hepatic side effects, as well as rare cases of abnormal immune responses such as pseudo- and hyperprogression.^43^

Efforts are underway to reduce the toxicity and increase the activity of these agents by co-administering them with one or more types of therapies (e.g., another immunotherapy or cytotoxic chemotherapy). The combination of PD-1/PD-L1 inhibitors with a CTLA-4 inhibitor has shown promise, as evidenced by the approval of nivolumab in combination with ipilimumab for the treatment of metastatic melanoma, advanced renal cell carcinoma and mismatch repair-deficient (dMMR) colorectal cancer.^44,45^ In addition, the combination of pembrolizumab with cytotoxic chemotherapy demonstrated clinical benefit, and was approved by the US Food and Drug Administration (FDA) for first-line treatment of metastatic non-small-cell lung cancer (NSCLC).^46^ The FDA has also approved pembrolizumab or avelumab in combination with axitinib, an inhibitor of vascular endothelial growth factor (VEGF) receptor, as a first-line treatment for advanced renal cell carcinoma.^44^ Atezolizumab in combination with chemotherapy was approved for advanced small-cell lung cancer^47^ and for certain women with advanced triple-negative breast cancer.^48^ In 98 clinical trials (*n* = 24,915) of PD-1 inhibitors, monotherapy was compared with combination therapy by indication and line of therapy, with combination therapy demonstrating increased objective response rates in 82.7% of the trials.^49^

## CD8^+^ T-cell (CAR T-cell) adoptive transfer

At the core of CD8^+^ T-cell engineering is the transduction of genes that specify and augment CD8^+^ T-cell functionality. Initially, CD8^+^ T-cell gene transduction was undertaken using retroviral vectors to clone tumour-specific TCRα and β chains, generating CD8^+^ T cells that specifically recognised tumour-associated antigens in an MHC-I-dependent manner. The process, however, was laborious and expensive. To bypass the isolation and expansion of autologous tumour-reactive CD8^+^ T cells, the use of bulk T cells from the peripheral blood for CAR T-cell manufacturing has been applied, and has revolutionised adoptive cell transfer in oncology.^50^

### The development of CARs

**Fig. 5:**
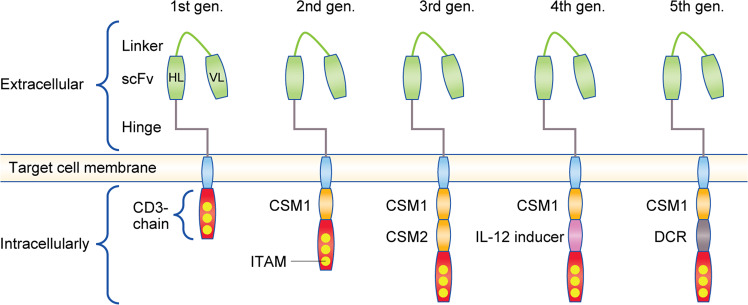
Development of chimaeric antigen receptors (CARs). The CAR construct comprises an antigen-binding scFv attached via a hinge to the CD3ζ signalling unit. Intracellularly, additional signalling units are attached to the CD3ζ chain. First-generation CARs contained only tyrosine-based activation motifs (ITAM) in the CD3 ζ-chain intracellular domain. Second-generation CARs include one co-stimulatory molecule, such as CD28 or 4-1BB, whereas third-generation CARs contain two co-stimulatory molecules, such as CD28 + 4-1BB (CD137). Fourth-generation CARs are based on second-generation CARs paired with a constitutively or inducibly expressed cytokine (e.g., IL-12) to further expand the T-cell population. Fifth-generation CARs are based on second-generation CARs with the addition of the intracellular domains of cytokine receptors (e.g., IL-2Rβ). CSM: co-stimulatory molecule, DCR: domain of cytokine receptors, IL-12: inducer activator of interleukin-12 transcription.

The CAR T-cell process involves the construction of a synthetic CAR^51^ by fusion of the single-chain variable fragment (scFv) of an antigen-specific immunoglobulin with an intracellular signalling domain, most often the transmembrane domain and endodomain of the CD3-ζ co-receptor, followed by the expansion of these autologous T cells and reinfusion back into the patient. A CAR can be designed to recognise any cancer cell-surface structure (protein, carbohydrate or glycolipid) independent of APCs and MHC presentation.^52^ First-generation CARs were constructed with an antigen-binding scFv attached via a hinge or spacer protein to the transmembrane and intracellular domains of CD3ζ (Fig. 5). The hinge determines target accessibility, with long hinges providing extra flexibility to access membrane-proximal antigens or complicated epitopes. In second- and third-generation CARs, a co-stimulatory signalling domain (often that of CD28 or CD137/4-1BB) was inserted between the transmembrane and signalling domains of the ζ chain to counteract T-cell anergy, which was seen with first-generation CARs, and to support T-cell expansion and persistence.^53^ Fourth- and fifth-generation CARs also include signalling domains for cytokine receptors, such as IL-12 or IL-18 (Fig. 5), to further expand the T-cell population without the associated toxicities of systemic interleukin administration.^54^

### Highlights and pitfalls of CAR T-cell therapy

Initially, CAR T-cell therapy was a game changer for the treatment of relapsed or refractory acute lymphoblastic leukaemia and diffuse large B-cell lymphomas, proving to be very successful in several clinical trials. CD-19 was selected as the CAR target due to its high expression in these conditions and very restricted expression in healthy tissues^55^ and, in 2017, the FDA approved two CD-19 CAR T-cell products (Kymriah and Yescarta) for these indications.^56^ To increase the potency of CAR T cells, patients are pre-conditioned with a lymphodepleting chemotherapy regimen, mainly cyclophosphamide and fludarabine, prior to adoptive T-cell transfer.^57^

However, despite impressive results, there have been important drawbacks, mainly due to the side effects caused by cytokine release (‘cytokine storm’ or ‘cytokine release syndrome’), leading to life-threatening immune hyperactivation and neurological toxicities.^58^ Cytokine-release syndrome is thought to be mediated mainly through the release of IL-1, and targeted anti-IL-1 therapy might reduce this side effect.^59^

Currently, projects using gene-editing tools, such as CRISPR/Cas9 to edit out genes that increase the risk of side effects, are under investigation.^52^ To minimise CAR T-cell toxicity and increase therapeutic efficacy, the use of synthetic Notch (SynNotch) receptors and bispecific T-cell engagers (BITEs) might provide ways to customise immune cell responses.^60^ SynNotch receptors have demonstrated specificity enhancement in CAR T-cell therapy.^61^ Combining the SynNotch receptor with a CAR is a simple and powerful way to induce the expression of toxic CARs to the tumour. The CAR can be designed to stay non-cytotoxic until the SynNotch receptor is engaged by antigens present in the local tumour microenvironment (TME). Such features may make the sequentially armed T cell a more effective and safer alternative for combinatorial antigen sensing.^62^

BITE therapy involves a polyclonal T-cell response, independent of MHC and TCR recognition and co-stimulation. Also, BITEs have the advantages of a low-molecular mass enabling tissue penetration and a relatively simple recombinant production process. In 2014, the first BITE (blinatumomab) gained FDA approval for acute lymphoblastic leukaemia.^63^

## The TME and immune-escape mechanisms

The common lack of response to immunotherapy in solid tumours can be explained by a number of factors, including irreversible CD8^+^ T-cell dysfunction, scarcity of antigens and/or mutations in the antigen-presentation machinery.^64^ CD8^+^ cells can also be excluded from, or trapped within, tumours by the dense, fibrotic extracellular matrix produced by cancer-associated fibroblasts (CAFs). Furthermore, to evade adaptive immune surveillance, tumour cells can downregulate the expression of MHC by up to 90% and increase the expression of immune-checkpoint ligands such as PD-L1, PD-L2,^65^ TIGIT and TIM-3, as well as transforming growth factor (TGF)-β and interleukins, all of which cause T-cell exhaustion.^66^ Some tumour cells can also downregulate or mutate essential enzymes, such as Janus kinases (JAK1 and JAK2). JAK mutations lower the ability of CD8^+^ T cells to recognise tumour cells and are implicated in primary resistance to PD-1 inhibitors.^67^ Therapy resistance can be correlated with increased expression of VEGF,^68^ which affects CD8^+^ T-cell functionality both directly and indirectly via the suppression of dendritic cell maturation and recruitment of suppressive cell populations.^69^

### CD8^+^ T-cell distribution within tumours

**Fig. 6:**
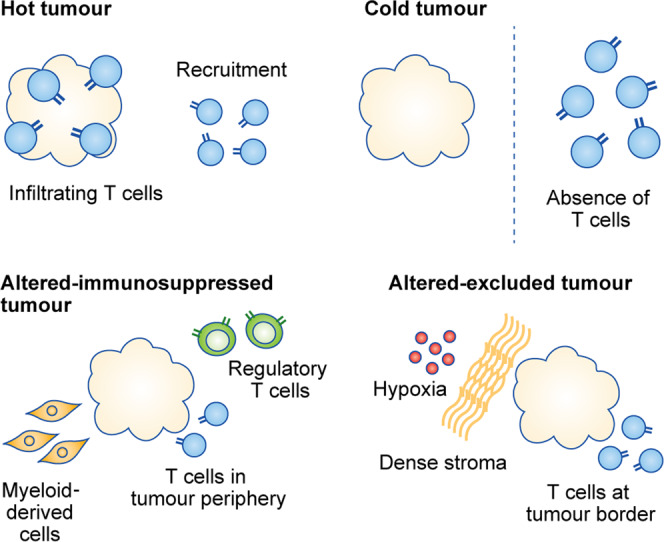
CD8^+^ T-cell distribution within tumours. In solid cancers, tumours can be classified as hot, cold, altered–immunosuppressed or altered–excluded tumours based on the degree of tumour CD8^+^ T-cell infiltration and the composition of the tumour microenvironment (TME). Hot tumours are characterised by high infiltration of CD8^+^ T cells and respond better to immune-enhancing therapies, whereas cold tumours are less likely to benefit from such treatments. Cold tumours are characterised by the absence of CD8^+^ T-cell infiltration, while altered–immunosuppressed tumours have sparse CD8^+^ T-cell infiltration, localised in the tumour periphery, and the presence of myeloid-derived suppressor cells and regulatory CD8^+^ T cells in the tumour tissue. Altered–excluded tumours are dominated by an abnormal vasculature (and consequent hypoxia) and a dense stroma, while CD8^+^ T cells are located at the border of these tumours.

A ‘cold’ tumour is a common resistance phenotype observed across solid cancers.^70^ The classification of hot and cold tumours relies partly on the degree and localisation of infiltrating CD8^+^ T cells, as well as the composition of the TME.^71^ Hot tumours are characterised by high infiltration of non-exhausted T cells, especially CD8^+^ T cells,^71^ whereas cold tumours lack T-cell infiltration and fail to induce T-cell priming (Fig. 6). In 2019, two new immunogenic categories of tumour were proposed by Galon and Bruni on this basis: altered–immunosuppressed and altered–excluded immune tumours.^71^ Altered–immunosuppressed tumours are characterised by a sparse infiltration of CD8^+^ T cells, which are typically located in the periphery of the tumour, and the presence of immune-suppressing cells, such as myeloid-derived suppressor cells and regulatory T cells.^71^ In altered–excluded immune tumours, CD8^+^ T cells are absent, and the TME is often dominated by the presence of a dense stroma and hypoxia, challenging the survival of immune cells.^71^ Cold, altered–immunosuppressed and immune–excluded tumours benefit less from immunotherapy and thus have a worse prognosis than hot tumours, which generally respond well.^71,72^ In a study of various cancers, the abundance of CD8^+^ T cells within a tumour was found to be the best predictive factor for the response to anti-PD-1/PD-L1 therapy.^72^

### Composition of the TME

Along with the degree and distribution of different CD8^+^ T-cell subsets, the composition of the TME is also of prognostic importance. Subverted immune cells, stromal cells and associated factors in the TME, especially accumulated CAFs, tumour-associated macrophages (TAMs), tumour-secreted factors (TSFs) and tumour-secreted exosomes (TSEs), contribute to CD8^+^ T-cell exhaustion.

Exosomes are extracellular vesicles containing genetic material, proteins and lipids produced by all types of cells with a role in intercellular communication; in the case of tumour cells, TSEs function as critical mediators of communication between cancer and immune cells.^73^ TSEs are considered major drivers of the formation of pre-metastatic niches (areas in a secondary organ that provide favourable conditions for metastasis) and mediate the reprogramming of target-cell transcriptomes to promote invasiveness and metastasis.^74,75^ They have been reported to drive bone marrow-derived cells (BMDCs) towards immunosuppressive, pro-tumorigenic and pro-angiogenic phenotypes, aiding in immune evasion by blocking dendritic cell maturation via induction of apoptosis and attraction of immunosuppressive regulatory T cells.^76^

TSFs include tumour necrosis factor, TGF-β and VEGF, which upregulate the expression of key molecules such as S100A8/9 (calprotectin), lysyl oxidases, fibronectin and metalloproteinases, and recruit large numbers of various cell types (e.g., regulatory T cells, mesenchymal stem cells, macrophages and neutrophils) to the TME. In addition, TSFs mobilise and attract immunosuppressive BMDCs while converting local stromal cells into pro-tumorigenic cells, such as the conversion of macrophages and fibroblasts into pro-tumorigenic TAMs and CAFs.^77^ TAM–CAF co-operation is a key determinant of the establishment of a cold tumour.

TAMs stimulate angiogenesis through VEGF-A secretion and promote immune suppression through TGF-β and IL-10 expression. Likewise, CAFs can stimulate angiogenesis and produce the dense intra-tumoural stroma mentioned earlier. Thus, the presence of TAMs and CAFs in a tumour indicates a TME hostile to lymphocytes, and is characteristic for an immunogenic cold tumour. TAMs and CAFs may co-operate to stimulate tumour progression, and the presence of both has also been linked to reduced survival.^78^

In addition, TAMs and TME mediators (e.g., exosomal PD-L1 and VEGF) inhibit the extravasation of CD8^+^ T cells from the circulatory system, as well as the replication and viability of CD8^+^ T cells within a tumour.^79^ In models of colorectal cancer and mesothelioma, the depletion of TAMs restored CD8^+^ cell infiltration and migration within tumours and improved the efficacy of anti-PD-1 immunotherapies.^79^

Even if CD8^+^ T cells manage to penetrate the fibrotic extracellular matrix tumour barrier, they need to upregulate the activity of metabolic pathways and remain activated under these demanding conditions, or they will acquire hyporesponsive phenotypes that cannot be rescued by stimulation.^80^ Furthermore, within the bulk of a tumour, CD8^+^ T cells need to actively navigate and seek out cancer cells—direct contact between T cells and cancer cells is decisive in determining the outcomes.^81^ Inadequate CD8^+^ T-cell navigation within a tumour represents a serious resistance mechanism: the amount and density of fibrosis, which is common in many carcinomas, is associated with resistance to chemotherapy and shortened disease-free survival.^82^ CAFs significantly inhibit the proliferation and differentiation of CD8^+^ T cells by inhibiting IL-2 production, which is essential for CD8^+^ T-cell function.^83^ Moreover, upregulation of FAS/FASL and PD-1/PD-L2 on T cells and CAFs, respectively, drives the dysfunction and death of CD8^+^ T cells, resulting in enhanced tumour cell viability.^84^

Even though tumours with high mutational loads and microsatellite instability favour CD8^+^ T-cell infiltration, the upregulation of WNT/β-catenin signalling correlates with the absence of CD8^+^ T-cell infiltration.^85^ β-Catenin is critical for transcription and proliferation in many types of human cancer. The WNT/β-catenin signalling pathway participates in the regulation of the NF-κB and TGF-β pathways and downregulates the expression of interferon regulatory factor 3, a transcription factor essential for the differentiation and maturation of immune cells, and is therefore instrumental for the exclusion of CD8^+^ T cells from the TME.^86^ In a multi-omic analysis of 1211 patients with colorectal cancer (several subtypes) and positive predictive biomarkers for immunotherapy, WNT/β-catenin signalling genes were significantly mutated and upregulated in all colorectal cancer subtypes and directly associated with treatment failure.^87^ By contrast, downregulation of β-catenin expression in colorectal cancer cell lines led to the increased production of anti-tumorigenic interferons and susceptibility to treatment.

### Conditions within the TME

To generate the energy required for effector functions, CD8^+^ T cells are highly dependent on the conditions within the TME, which is mostly dominated by a lack of nutrients, an abnormal vasculature, high interstitial pressure, hypoxia and acidity, and thus is hostile to CD8^+^ T cells.

During hypoxia, mammalian cells secrete hypoxia-inducible factor (HIF), which facilitates continued ATP production in an oxygen-independent manner. HIF also mobilises BMDCs, stimulates the release of both TSFs and TSEs and increases the production of chemokines and VEGF.^88^

Hypoxia promotes immunosuppression and induces epithelial–mesenchymal transition by upregulating the expression of transcriptional repressors of E cadherin.^89,90^ In cancer cells, hypoxia and autophagy have a variety of complicated and competing roles. Hypoxia can slow the rate of cell death and provide cancer cells with an opportunity to survive and maintain growth.^91^ The surviving cells might then develop genomic instability, which further enhances tumorigenesis in the absence of cell death.^92^ Clinical interventions to alter autophagy in cancer are being developed.^93^

Autophagy can stimulate tumour antigen cross-presentation, supporting the fact that an improved tumour-immune response and autophagy inhibition could potentially interfere with this process. However, autophagy inhibition may also enhance antitumour-immune responses as both a strong inhibition and a strong induction of autophagy can lead to cancer cell death.^94^

Many interventions currently used are altering autophagy in cancer patients, and the focus is on how to maximise a potential benefit. Oncologists have remained excited, particularly around the opportunity for the use of autophagy inhibitors in RAS-driven cancers, which account for more than 30% of cancers.^95^

## Conclusions and discussion

The era of biological cancer therapy emerged in 1968 with the first successful non-specific allogeneic stem cell transplant.^96^ In the 1990s, IFN-α was approved for the treatment of hairy cell leukaemia, and the first monoclonal antibody, trastuzumab, was approved for the treatment of patients with breast cancer caused by overexpression of HER2. These approvals were soon followed by the release of cetuximab (anti-epidermal growth factor receptor) in 2004 and bevacizumab (anti-VEGFR) in 2006.

Currently, we are witnessing another major step forward with the introduction of immune-checkpoint inhibitors. In 2011, the FDA approved the first of a new generation of monoclonal antibodies designed to boost the adaptive immune response by targeting CD8^+^ T-cell checkpoints and revitalising exhausted CD8^+^ T cells. The product was an anti-CTLA-4 antibody (ipilimumab) for the treatment of metastatic melanoma. Ipilimumab was followed by PD-1-targeted antibodies (pembrolizumab and nivolumab) in 2014 and an anti-PD-L1 antibody (atezolizumab) in 2016 for advanced melanoma, squamous cell lung cancer, small-cell lung cancer, Hodgkin’s lymphoma and bladder cancer. Immune-checkpoint inhibitor therapy is currently approved for use in a wide range of tumours, including melanoma, NSCLC, renal cell cancer, Merkel cell cancer, Hodgkin’s lymphoma, urothelial cancer and dMMR/microsatellite instability-high colorectal tumours, and the indications are rapidly expanding. Presently, there are 3394 immuno-oncology therapies in the global development pipeline, with 1287 of these in clinical trials.^97^ In the US, the percentage of patients with cancer eligible for checkpoint inhibitor treatment increased from an estimated 1.5% in 2011 to almost 44% in 2018. However, the overall response rate of 13% in eligible patients is lower than that hoped for and might translate into serious overtreatment with unnecessary risks of severe side effects.^42^

The one-shot cures of lymphomas and leukaemias delivered by CAR T-cells in early clinical trials were unexpected and have resulted in major research activities and resource allocation. CAR T-cell therapy is a costly and highly resource-consuming process that is currently limited to the indications of children and young adults with acute lymphoblastic leukaemia for whom conventional chemotherapy has been ineffective. Eliminating the need for viral vectors will simplify processes and reduce costs, and it is very likely that immune cell editing using CRISPR/Cas9 technology will be at the forefront of the cell-transfer therapy revolution as new generations of CARs are developed and introduced in the clinic and investigated for the experimental treatment of solid cancers. The development of ‘off-the-shelf’ CAR T-cell products for MHC-matched patients requires therapies that target hot-spot driver-gene-mutation-derived neoantigens, e.g., KRAS or p53.^98^ Among the major limitations of CAR T-cell treatment, CAR T cells recognise only surface proteins and are not entirely exclusive to tumour-specific cell-surface proteins, and thus, there is a risk of cross-reactivity with normal tissue proteins, which could lead to serious adverse events. However, CAR T-cell therapy could be combined with immune-checkpoint inhibitors, interleukin administration, NK cells, radiotherapy, epigenetic drugs and cytotoxic chemotherapy. Chemotherapy was previously thought to be solely immunosuppressive, but data show that the majority of chemotherapeutic drugs have immunostimulatory properties, either by inhibiting immunosuppressive cells and/or activating effector cells, or by increasing immunogenicity and T-cell infiltration.^99^ Indeed, the combination of immune-checkpoint blockade with other anticancer treatments has demonstrated^100^ remarkable success in various cancers that do not respond to checkpoint inhibitor monotherapy.

Currently, most solid tumours are surgically removed, and evidence points to the ability of inflammatory, endocrine and neural responses in the perioperative period to cause immunosuppression and promote the survival and dissemination of tumour cells, as well as activation of dormant micrometastases. A large-magnitude surgical stress response is correlated with poor long-term oncological outcomes,^101^ and is mainly attributed to the impairment of cytotoxic immune cells.^102,103^ However, the armamentarium of cancer treatment is rapidly increasing, and personalised treatment in terms of combination therapy directed specifically at the central target in cancer biology, e.g., immunotherapies customised and directed at patient-specific molecular mutations, is a promising treatment strategy. Cutting-edge synthetic biology using tailored receptors, such as SynNotch receptors and BITEs, will take the development of immunotherapy even further, and combinatory regimens, including genetically modified immune cells and other anticancer interventions, such as immune-checkpoint inhibitor therapy and chemotherapy, will presumably have major impacts on the treatment of cancers.

## Data Availability

Not applicable.
